# Impact of the functionalization onto structure transformation and gas adsorption of MIL-68(In)

**DOI:** 10.1098/rsos.181378

**Published:** 2018-12-12

**Authors:** Lei Wu, Weifeng Wang, Rong Liu, Gang Wu, Huaxin Chen

**Affiliations:** 1 Polymer Materials and Engineering Department, School of Materials Science and Engineering, Chang'an University, Xi'an 710064, People's Republic of China; 2 Engineering Research Center of Transportation Materials Ministry of Education, Chang'an University, Xi'an 710064, People's Republic of China; 3 State Key Laboratory of Inorganic Synthesis and Preparative Chemistry, College of Chemistry, Jilin University, Changchun 130012, People's Republic of China

**Keywords:** MOFs, MIL-68(In)_X, functionalization, structure transformation, gas adsorption

## Abstract

A series of functionalization –NH_2_, –Br and –NO_2_ has been performed on MIL-68(In) material in order to improve the porosity features of the pristine material. The functional groups grafted onto the ligand and the molar ratios of the ingredient indicate a profound influence on product formation. With the incremental amount of metal source, product structures undergo the transformation from MIL-68 to MIL-53 or QMOF-2. The situation is different depending on the variation of the ligands. Gas (N_2_, Ar, H_2_ and CO_2_) adsorption–desorption isotherms were systematically investigated to explore the impact of the functionalization on the porous prototypical framework. Comparison of adsorption behaviour of N_2_ and Ar indicates that the polar molecule exhibits striking interaction to N_2_ molecule, which has a considerable quadrupole moment. Therefore, as a probe molecule, Ar with no quadrupole moment is more suitable to characterize the surface area with the polar groups. Meanwhile, Ar adsorption result confirms that the negative influence on the surface area stems from the size of the substituting groups. The uptake of H_2_ and CO_2_ indicates that the introduction of appropriate polar organic groups can effectively enhance the adsorption enthalpy of relative gases and improve the gas adsorption capacity apparently at low pressure. The introduction of –NO_2_ is in favour of improving the H_2_ adsorption capacity, while the grafted –NH_2_ groups can most effectively enhance the CO_2_ adsorption capacity.

## 1. Introduction

In the last two decades, metal-organic frameworks (MOFs) have developed as promising candidates for adsorption of gas, such as H_2_, CO_2_, CH_4_, owing to their ultra-high porosity, large surface area, well-defined open channels and structural diversity [1–9]. However, practical and industrial applications of those materials in realistic and non-ideal environments need to meet more rigorous requirements such as higher thermal stability and higher adsorption capacity. So, it is necessary to enhance the performance of the MOFs. It is experimentally or computationally demonstrated that functionalization of the porous MOFs with the appropriate substituents is an effective approach for performance optimization, such as introducing the metal unsaturated site (Li^+^, Cu^2+^), post-synthetic approaches or graft functional substance onto a coordinative unsaturated framework or the use of ligands with functional group to generate functionalized MOFs [10–14]. Zhang *et al*. made 24 MOF-177 structures with different functional groups on the triangular BTB linker, having one or more functionalities [15]. It has been found that the position of the functional groups on the BTB unit shows the selection for the framework net. Meanwhile, the mixing of functionalities (–H, –NH_2_, –C_4_H_4_ and –NO_2_) into MOF-177 leads to the enhancement of hydrogen uptake by 25%. Xia *et al*. study the effect of functional groups on the hydrogen storage properties of MOF-808-X (X = –OH, –NO_2_, –CH_3_, –CN, –I) with the GCMC method [16]. That the simulated H_2_ uptakes isosteric heat at 77 K indicates that all these substituents have a favourable impact on the hydrogen storage capacity, and –CN is the most promising substituent to improve H_2_ uptake. Frysali *et al*. reported that the OSO_3_H functional group possesses the highest binding energy with CO_2_ among the 14 selected functional groups with RI-MP2/aug-cc-pVTZ method [17]. Depending on the functional group, CO_2_ acts as Lewis acid and Lewis base simultaneously. This cooperative effect stabilizes the formed complex and affects the location of CO_2_ in the optimized geometries. Mu *et al*. study the effect of the chemical properties of nine organic linkers on CO_2_/CH_4_ mixture separation in MOFs with a hierarchical multiscale approach combining GCMC simulation and DFT calculation [18]. The results show that the organic linkers decorated with the electron-donating groups can strengthen the distribution of the electrostatic field in the pores of MOFs and greatly enhance the adsorption selectivity of the CO_2_/CH_4_ mixture. In addition, this work also demonstrates that the steric hindrance indicates the negative effects on the separation behaviour. Despite extensive research work, there is plenty of room to explore the impact of the functionalization onto the properties of MOFs due to the rapidly growing species of MOFs.

Motivated by it, this work is devoted to improving the porosity of the prototypical material by implementing different functional groups. To realize functionalization, high stability of the prototype framework and the channels with appropriate dimension should be required. Therefore, MIL-68(In) with high thermal stability and high BET surface area, reported by Volkringer and co-workers, is the priority as the prototype structure. There exist two kinds of the channels with sufficient diameter openings (approx. 6.0 and approx. 16 Å) in the skeleton which makes it possible to further introduce the specific substituent groups into the pore to implement channel functionalization [19]. Meanwhile, –NH_2_, –Br and –NO_2_ with different dimension and polarity were selected as functional groups to probe the influence onto the gas adsorption behaviour of the prototype MIL-68(In).

But, related to MIL-type materials, some interesting structural transformations have been reported due to different important factors, such as pH, time, temperature and guest molecules. Perea-Cachero *et al.* described a method for the reversible crystalline transformation between MIL-53(Al) and MIL-68(Al), which had higher thermal stability and crystallinity than the as-synthesized MIL-68(Al) [20]. Carson *et al*. reported topological isomers MIL-101(V) and MIL-88B(V) converted into MIL-47(V) by a thermal treatment (200°C) [21]. Liédana *et al*. demonstrated that caffeine played the role of a structure directing agent or template, which led NH_2_-MIL-88B(Fe) to undergo a reversible transformation into NH_2_-MIL-53(Fe) [22]. During our synthesis process, a series of regular crystalline structural transformations among MIL-68(In)_X, MIL-53(In)_X and QMOF-2 were observed along with the adjustment of the reactants. MIL-53(In) (sra topology) is polymorph with MIL-68(In), constructed from infinite chains of inorganic InO_4_(OH)_2_ octahedra linked by the apical trans hydroxyl functions with the flexible framework and unique rhombic cavities (*ca* 8.5 Å) [23]. QMOF-2 constructed from distorted pseudo-tetrahedral coordinated indium centres linked by carboxylate ligands with the high symmetry of the *β*-quartz network [24]. To the best of our knowledge, no attention, however, has ever been paid to study the impact of the functionalization onto the structural transformation of these MOFs to date, which is one main aspect of our study.

In this work, powder X-ray diffraction analysis, IR spectroscopy, ^1^H NMR spectroscopy, TG analysis and the gas (N_2_, Ar, H_2_, CO_2_) sorption behaviours were fully investigated to determine the influence of the functionalization on the prototype MIL-68(In). The results highlight the effectiveness of the functionalization of the porous MOFs to improve the porous characteristics. Notably, our current work provides an insight into the dramatical influence of the functional groups grafted on the ligand as well as the molar ratios of the ingredient onto the structural formations of MIL-type materials.

## 2. Experimental section

### 2.1. Reagents

All reagents and solvents received from commercial suppliers were used without further purification (indium nitrate hydrate, In(NO_3_)_3_·*x*H_2_O, Alfa Aesar, 99%; terephthalic acid (H_2_BDC), Alfa Aesar, 98%; 2-Aminoterephthalic acid (H_2_BDC-NH_2_), Alfa Aesar, 99%; 2-Bromoterephthalic acid (H_2_BDC-Br), Alfa Aesar, 97%; Nitroterephthalic acid (H_2_BDC-NO_2_), Alfa Aesar, 99%; *N*,*N*-Dimethylformamide (DMF), Aldrich, greater than 99%; anhydrous ethanol, Prolabo, 99.8%; anhydrous methanol, Aldrich, 99.9%).

### 2.2. Synthesis

Experiments on the investigation of structure transformation rule were carried out in accordance with the following strategy. A mixture of In(NO_3_)_3_·*x*H_2_O (*x* mmol), H_2_BDC-NH_2_ (*y* mmol) and DMF (1.24 ml, 17.30 mmol) was placed in a Teflon-lined home-made stainless steel autoclave (2.40 ml) and heated for 5 or 24 h at 125°C in an oven. The resulting powder was filtered and then washed with DMF. Metal/ligand molar ratios were modulated in three different synthesis systems, which were respectively M : L = 3 : 1, 1 : 1, 1 : 3 or 1 : 6. The MIL-68(In) sample was prepared according to the reported recipe [19].

### 2.3. Activation process

To remove the guest molecules, an activation process was performed on each crude sample of MIL-68(In)_X before gas adsorption measurements. The as-synthesized samples are soaked in the organic solvents with low boiling point (MIL-68(In)_Br with anhydrous ethanol, MIL-68(In)_NH_2_ and MIL-68(In)_NO_2_ with anhydrous methanol) for 3 days and the solvents were changed twice a day. After the filtration, each sample was heated (MIL-68(In)_Br at 150°C, MIL-68(In)_NH_2_ and MIL-68(In)_NO_2_ at 200°C) under the flow of N_2_ for 5 h. Before the measurement, the samples were degassed again by using the ‘degas’ function of the surface area analyser at a corresponding temperature for 12 h in vacuum.

### 2.4. Techniques of characterization

The IR spectra were recorded (400–4000 cm^–1^ region) on a SHIMADZU IRAffinity-1 Fourier-transform infrared spectrometer by using the KBr pellets method. The PXRD patterns were carried out on an STOE STADI-P diffractometer equipped with a curved germanium (111) primary monochromator and a linear position-sensitive detector using Cu K*α*1 radiation, *λ* = 1.5406 Å. The patterns were registered in the 3–50° 2*θ* range with a scanning step of 0.12° s^–1^. The size and the morphology of the crystals were determined by scanning electron microscopy (SEM) using a Philips XL 30 FEG microscope. TGA was performed under nitrogen with a heating rate of 5°C min^−1^ up to 800°C using a Perkin–Elmer TGA 7 thermogravimetric analyser. ^1^HNMR spectra were carried out on a Bruker 400 UltraShield™ by using tetramethylsilane as standard.

Argon adsorption isotherms were performed on a Quantachrome Antosorb-IQ-C apparatus. Ar isotherms at 87 K were measured in a liquid argon bath using an 87 K sensor. Nitrogen, hydrogen and carbon dioxide adsorption isotherms were performed on a Micromeritics Tristar II 3020 apparatus. H_2_ isotherms at 77 K were measured in a liquid nitrogen bath using a 77 K sensor, while H_2_ isotherms at 87 K in a liquid argon bath using an 87 K sensor. CO_2_ isotherms at 273 K were measured in an ice-water bath using a 273 K sensor, while CO_2_ isotherms at 298 K were measured at room temperature controlled by central air-conditioning.

## 3. Results and discussion

### 3.1. Structure transformation analysis

**Figure 1.**
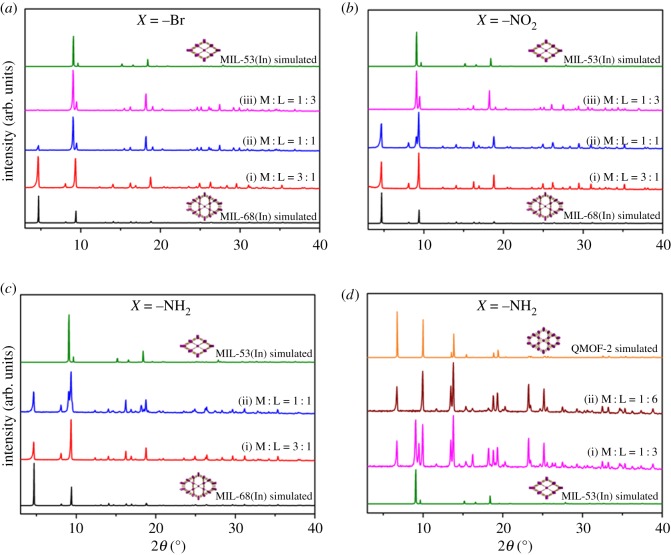
PXRD patterns for the samples synthesized from different synthesis systems: organic ligand is (*a*) H_2_BDC-Br; (*b*) H_2_BDC- NO_2_ and (*c*) H_2_BDC-NH_2_ (M : L = 3 : 1, 1 : 1) and (*d*) H_2_BDC-NH_2_ (M : L = 1 : 3, 1 : 6).

**Figure 2.**
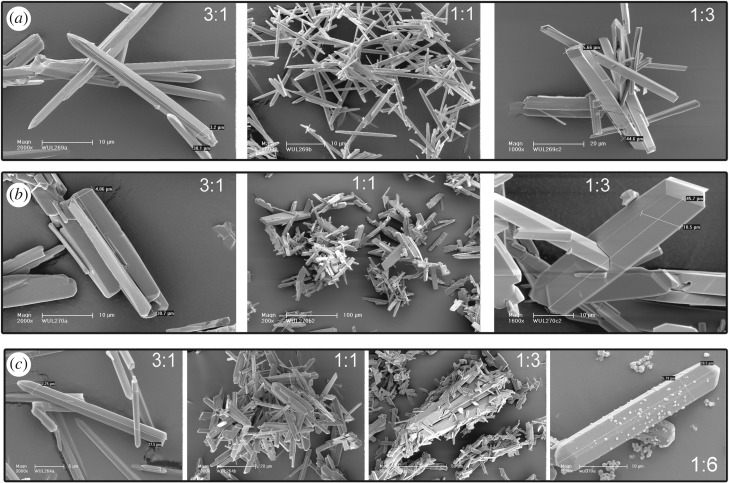
SEM photographs of the samples synthesized from different synthesis systems: organic ligand is (*a*) H_2_BDC-Br; (*b*) H_2_BDC- NO_2_ and (*c*) H_2_BDC-NH_2_.

Based on the pre-experimental studies, solvent, temperature and time were fixed in three different reaction systems, while the metal/ligand molar ratio was variable. Because the crystal size was not appropriate for performing SCXRD, PXRD was used to characterize the crystalline structure of the samples. The PXRD pattern is depicted in figure 1. Corresponding morphological changes were tracked by SEM, as shown in figure 2.

The PXRD results show that the molar ratios of the ingredients have a profound influence on product formation. The similar variation trend can be observed in the presence of H_2_BDC_Br and H_2_BDC_NO_2_. Exclusive MIL-68(In)_X is formed with an excess amount of In^3+^ (M : L = 3 : 1). While with M : L = 1 : 1, the Bragg peak assigned to MIL-53(In)_X is discovered in the corresponding PXRD spectra, which indicates that structural transformation is discovered as the amount of the ligands increases. With M : L = 1 : 3, absolute MIL-53(In)_X is observed in the presence of excess ligands. The morphological transformation, from hexagonal needle shape to rhombic block shape, can be clearly observed in the corresponding SEM images (figure 2*a,b*). The analogous crystalline-state transformation between MIL-53(Al) and MIL-68(Al) was reported by Perea-Cachero and co-workers [20]. The transformation mechanism is regarded as the exchange or removal of guest molecules from the pores.

However, the PXRD results show that dissimilar structural transformation occurred with the incremental amount of H_2_BDC_NH_2_. As the amount of the ligand switched from 3 : 1 to 1 : 1, the structural transformation is similar to another two synthesis systems from MIL-68(In)_X to the mixed phase (MIL-68(In)_X and MIL-53(In)_X). But, the Bragg peak assigned to QMOF-2 can be obviously observed in the PXRD spectra of M : L = 1 : 3, which indicates that disparate structural transformation process is taking place with an excess amount of H_2_BDC_NH_2_. Choi and co-workers reported the analogous structural transformation from MIL-68(In) to QMOF-2, and verified that MIL-68(In) is an initial kinetic product and QMOF-2 is a final thermodynamic product of In^3+^ with an excess amount of H_2_BDC [25]. To verify the tenability of this conclusion in In^3+^ and H_2_BDC_NH_2_ system, the amount of the ligand was continuously increased to M : L = 1 : 6, and the reaction time was prolonged to 24 h. The PXRD result validates that exclusive QMOF-2 is formed. Seen from the corresponding SEM images, the morphological transformation goes from hexagonal needle shape to hexagonal needle shape mixed with rhombic block shape, and then to rhombic block shape mixed with two-based pointed hexagonal rod shape, finally to two-based pointed hexagonal rod shape (figure 2*c*).

It is interesting to note that distinguishing crystal structure formations occurred in the three systems, with the same M : L = 1 : 3. Referring to the three pristine crystallographic frameworks, in MIL-68 and MIL-53 In^3+^ adopted in hexa-coordination with six O atoms, four of which come from four monodentate carboxyl ligands, two from *µ*-connecting OH groups (electronic supplementary material, figure S1*a*). Whereas, in QMOF-2 In^3+^ adopted in octa-coordination with eight O atoms come from four chelating bidentate carboxyl ligands (electronic supplementary material, figure S1*b*). Therefore, the carboxylic ligands are coordinated to metal centres more steadily and closely in the crystallographic configuration of QMOF-2 relatively. Compared to the NH_2_ group, Br and NO_2_ groups are with larger dimensions leading to the larger space resistance. The higher energy barrier needs to be overcome, which makes it more difficult to the formation of final thermodynamic product QMOF-2 with H_2_BDC_Br and H_2_BDC_NO_2_.

### 3.2. Structure description of MIL-68(In)_X

The unit cell parameters of MIL-68(In)_X were determined from PXRD using DICVOL4 algorithm [26]. The comparison of the lattice parameters of MIL-68(In)_X and MIL-68(In) is shown in table 1, which exhibits the minimum variation among the four structures. Meanwhile, the perfect fit between PXRD pattern of as-synthesized samples and simulated spectrum of MIL-68(In) shows the insertion of the substituting groups does not lead to obvious structure variation (electronic supplementary material, figure S2). The Kagomé-like MIL-68(In) prototype framework has been sustained after the decoration of different substituting groups.

**Table 1.** Unit cell dimensions of MIL-68(In) [19] and MIL-68(In)_X.

func.	*a* (Å)	*b* (Å)	*c* (Å)	*V* (Å^3^)	system	space	*F* (30)
—	21.773	37.677	7.233	5933.8	orthorhombic	*Cmcm*	—
–NH_2_	37.804	21.782	7.215	5941.2	orthorhombic	*Cmcm*	37.0
–Br	37.716	21.824	6.359	5162.4	orthorhombic	*Cmcm*	27.9
–NO_2_	37.713	21.817	7.337	5984.6	orthorhombic	*Cmcm*	34.5

### 3.3. Gas adsorption measurements of MIL-68(In)_X

#### 3.3.1. N_2_ adsorption measurement

**Figure 3.**
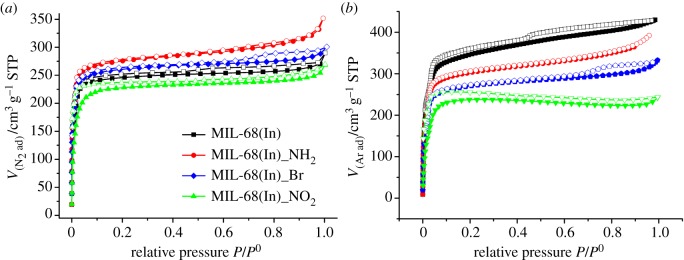
(*a*) At 77 K, the N_2_ sorption isotherms and (*b*) at 87 K, the Ar sorption isotherms of MIL-68(In) (black); MIL-68(In)_NH_2_ (red); MIL-68(In)_Br (blue); MIL-68(In)_NO_2_ (green) (adsorption, solid; desorption, empty).

N_2_ adsorption measurements at 77 K were performed on the activated samples to investigate the porosity of MIL-68(In)_X materials, and the same measurement has been done to MIL-68(In) for comparison. As shown in figure 3*a*, all of the N_2_ sorption isotherms are of type I isotherm characteristic of microporous solid with a sharp uptake in the low pressure region (10^–5^ to 10^–1^ atm). The comparison of N_2_ sorption results between MIL-68(In)_X and MIL-68(In) evidences that the introduction of –NH_2_ and –Br groups enlarges the surface area compared to prototype MIL-68(In) except for –NO_2_ group. The values of BET and Langmuir surface areas reach 1230 and 1288 m^2^ g^–1^ (MIL-68(In)_NH_2_); 1040 and 1152 m^2^ g^–1^ (MIL-68(In)_Br); 1028 and 1120 m^2^ g^–1^ (MIL-68(In)) and 954 and 1072 m^2^ g^–1^ (MIL-68(In)_NO_2_).

PXRD analysis demonstrates that the skeleton is sustained without the obvious variation after modification; however, the emplacement of the substituting groups leads to the different surface area for MIL-68(In)_X. We analyse that the improved N_2_ adsorption capacity generating from the interactions of polar groups–N_2_ molecule, which has a considerable quadrupole moment (table 2). Thus, the surface area calculated from the N_2_ adsorption quantity results in the higher value instead of the actual one.

**Table 2.** Quadruple moment of four selected gases [27].

gas	N_2_	Ar	H_2_	CO_2_
quadruple moment (×10^26^ emu cm^−2^)	1.52	0.00	0.66	4.30

#### 3.3.2. Ar adsorption measurement

To verify our conjecture and to obtain the actual surface area and pore volume, argon with zero quadrupole moment was chosen to be the probe molecule. And the adsorption result is shown in figure 3*b*. In contrast to the N_2_ adsorption result, that of MIL-68(In) shows the highest sorption capacity, which corresponds to the values of BET and Langmuir surface area of 1271 and 1378 m^2^ g^–1^, respectively. The Ar uptake of MIL-68(In)_X varied in the sequence of MIL-68(In)_NH_2_>MIL-68(In)_Br > MIL-68(In)_NO_2_. This order is in agreement with the size of the polar functional groups. BET and Langmuir surface area are 1108 and 1247 m^2^ g^–1^(MIL-68(In)_NH_2_), 1073 and 1127 m^2^ g^–1^ (MIL-68(In)_Br) and 987 and 1055 m^2^ g^–1^ (MIL-68(In)_NO_2_**)**. The result confirms that the influence of the size of the substituting groups on the surface area cannot be ignored [28–30]. Larger groups lead to more loss in the surface area or pore volume.

The results of N_2_ and Ar adsorption are summarized in table 3 for comparison. When the gas molecule is used to probe the surface area and pore volume of porous materials, the feature of the probe molecules and the pore wall of the porous materials cannot be ignored. The interaction between gas molecules and the pore wall can lead to the deformation of the probe molecules and then affect the gas adsorption capacity, which generates the deviation of the calculated surface area from the actual value. Such influence was proved to be evident in the case that N_2_ molecules with a considerable quadrupole moment were used to probe the surface area of the porous material with polar pore wall.

**Table 3.** Results of N_2_ and Ar adsorption measurement for MIL-68(In) and MIL-68(In)_X.

	N_2_			Ar		
compound	uptake (cm^3^ g^–1^)	*S*_BET_ (m^2^ g^–1^)	*S*_Langmiur_ (m^2^ g^–1^)	uptake (cm^3^ g^–1^)	*S*_BET_ (m^2^ g^–1^)	*S*_Langmiur_ (m^2^ g^–1^)
MIL-68(In)	289.7	1028	1120	430.0	1272	1378
MIL-68(In)_NH_2_	351.7	1230	1288	364.7	1108	1247
MIL-68(In)_Br	300.8	1040	1152	332.7	1073	1127
MIL-68(In)_NO_2_	268.9	954	1072	243.5	987	1055

#### 3.3.3. Calculation method of adsorption enthalpy

To further understand H_2_ and CO_2_ adsorption behaviour of MIL-68(In)_X, the coverage-dependent isosteric heat of adsorption (hereinafter *Q*_st_ for short) was determined. At first, the combined isotherm data were modelled with a virial-type expression [31,32],

3.1 $$\ln P=\ln N+\frac{1}{T}\sum_{i=0}^{m}a_{i}N^{i}+\sum_{i=0}^{n}b_{i}N^{i},$$

where *a_i_* and *b_i_* are independent parameters, *P* is the pressure (mmol g^–1^), *N* is the uptake amount (mmol g**^–^**^1^), *T* is the temperature (*K*), and *m* and *n* determine the number of terms required to adequately describe the isotherm, in all cases, *m* ≤ 6 and *n* ≤ 3. Then from the results from the upper fitting, the independent parameter *a_i_* was used to calculate the isosteric heat of adsorption with the following expression:

3.2 $$Q_{\mathrm{st}}=-R\sum_{i=0}^{m}a_{i}N^{i},$$

where *R* is the universal gas constant of 8.3147 J K**^–^**^1^ mol**^–^**^1^.

#### 3.3.4. H_2_ adsorption measurement

**Figure 4.**
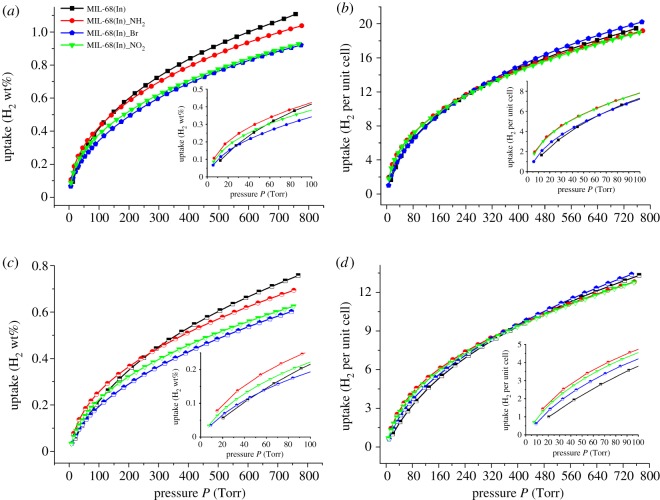
At 77 and 87 K, H_2_ sorption isotherms of MIL-68(In) (black); MIL-68(In)_NH_2_ (red); MIL-68(In)_Br (blue) and MIL-68(In)_NO_2_ (green) (*a*) in gravimetric percentage (77 K); (*b*) normalized per unit cell (77 K); (*c*) in gravimetric percentage (87 K) and (*d*) normalized per unit cell (87 K). (The inset is an enlargement of the low pressure region of the H_2_ isotherms.)

The H_2_ sorption isotherms of MIL-68(In) and MIL-68(In)_X at 77 and 87 K are depicted in figure 4. At 77 K and 1 atm (760 Torr), MIL-68(In) and MIL-68(In)_X exhibit the uptake of 1.11, 1.04, 0.92 and 0.93 wt%, respectively (figure 4*a*). At 87 K and 1 atm (760 Torr), the hydrogen uptake of aforementioned compounds is about 0.76 wt%, 0.69 wt%, 0.60 wt% and 0.62 wt%, respectively (figure 4*c*). Given the distinguishing molecular weight due to the different substituting groups, the molecules per unit cell are calculated to analyse the influence of the substituting groups on H_2_ adsorption. Seen from figure 4*b*, at 77 K and 1 atm (760 Torr), the H_2_ uptake of MIL-68(In) and MIL-68(In)_X is equivalent to about 19.5, 19.2, 20.2 and 19.0 H_2_ molecules per unit cell, respectively. Meanwhile, at 87 K and 1 atm (760 Torr), it corresponds to 13.3, 12.8, 13.4 and 12.8 H_2_ molecules per unit cell (figure 4*d*). The comparison result shows the subequal molecules per unit cell. However, considering the reductive surface area of the functionalized materials, the modification of organic groups has a positive influence on the capacity of H_2_ uptake, which is obviously reflected at low pressure. As shown in the inset of figure 4, the functionalized materials indicate higher or more rapid H_2_ absorption capacity.

**Figure 5.**
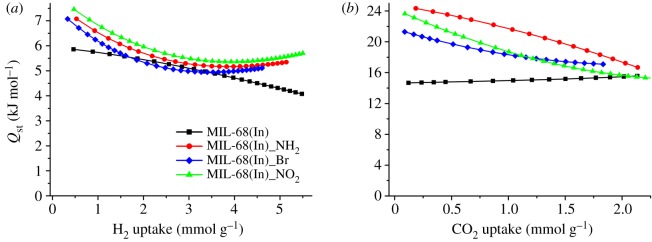
(*a*) H_2_ adsorption enthalpy, (*b*) CO_2_ adsorption enthalpy of MIL-68(In) (black); MIL-68(In)_NH_2_ (red); MIL-68(In)_Br (blue) and MIL-68(In)_NO_2_ (green).

To further understand the H_2_ adsorption behaviour, the H_2_ *Q*_st_ of each sample was determined from fits of 77 and 87 K isotherms, as shown in figure 5*a*. The *Q*_st_ of the functionalized materials shows the similar variation tendency. At the onset of the adsorption, the *Q*_st_ reaches around 7.00 kJ mol^–1^, and then decreases monotonically to approximate 5.20 kJ mol^–1^ at higher H_2_ pressures and remains steady at this value throughout the adsorption process. However, the maximum *Q*_st_ of MIL-68(In) is 5.86 kJ mol^–1^ at low coverage, and then decreases to 4.07 kJ mol^–1^ as the H_2_ loading increases. The comparison of the low-coverage *Q*_st_ between the functionalized materials and MIL-68(In) demonstrates that appropriate functionalization can contribute to the improved H_2_ *Q*_st_, which implies the existence of the excess interaction between adsorbent and adsorbate generated from the organic groups along the pore wall. It is proved that functionalization of benzene with an electron-donating group such as –NH_2_ could enhance the interaction between H_2_ and the phenyl rings regardless of the centroid or perpendicular direction. Although the affinity between H_2_ and the phenyl rings may be weakened by the electron-withdrawing groups (–Br and –NO_2_), it can be greatly enhanced due to the constrictions of the strong polarity of the two substituting groups, which can well make up for the former loss [33,34]. With the pore surface being occupied, the *Q*_st_ gradually decreases to a constant.

To facilitate the contrast, the H_2_ adsorption results of MIL-68(In) and MIL-68(In)_X are listed in table 4. H_2_ uptake of the isomorphic MIL-68(In)_X is dominated by the surface area or pore volume at high pressure. However, the introduction of polar organic groups can effectively enhance the H_2_ *Q*_st_, especially –NO_2_ and –NH_2_, thus effectively improving the H_2_ adsorption capacity at low pressure.

**Table 4.** Results of H_2_ adsorption measurement for MIL-68(In) and MIL-68_X.

	77 K		87 K		*Q*_st_ (mmol g^–1^)
compound	wt%	mole/unit cell	wt%	mole/unit cell	
MIL-68(In)	1.11	19.5	0.76	13.3	5.86
MIL-68(In)_NH_2_	1.04	19.2	0.69	12.8	7.08
MIL-68(In)_Br	0.92	20.2	0.60	13.4	7.07
MIL-68(In)_NO_2_	0.93	19.0	0.62	12.8	7.41

#### 3.3.5. CO_2_ adsorption measurement

**Figure 6.**
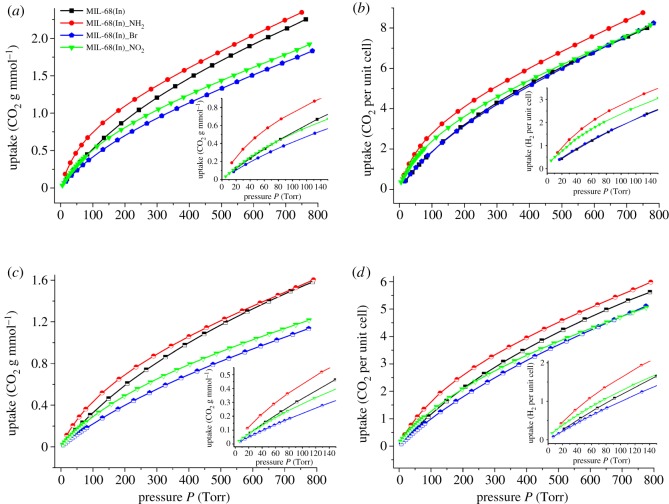
At 273 and 298 K, CO_2_ sorption isotherms of MIL-68(In) (black); MIL-68(In)_NH_2_ (red); MIL-68(In)_Br (blue) and MIL-68(In)_NO_2_ (green) (*a*) in gravimetric percentage (273 K); (*b*) normalized per unit cell (273 K); (*c*) in gravimetric percentage (298 K) and (*d*) normalized per unit cell (298 K).

The CO_2_ adsorption isotherms of MIL-68(In) and MIL-68(In)_X at 273 and 298 K are depicted in figure 6. At 273 K and 1 atm (760 Torr), MIL-68(In) and MIL-68(In)_X exhibit the uptake of 2.25, 2.35, 1.83 and 1.92 mmol g^–1^, respectively (figure 6*a*). And at 298 K and 1 atm (760 Torr), the CO_2_ uptake of aforementioned compounds is about 1.58, 1.60, 1.14 and 1.22 mmol g^–1^ (figure 6*c*). As mentioned above, in order to avoid the influence generated from the distinguishing molecular weight due to the different substituting groups, the molecules per unit cell are calculated to analyse the effect of the substituting groups on the CO_2_ adsorption. As shown in figure 6*b*, at 273 K and 1 atm (760 Torr), the uptake of MIL-68(In) and MIL-68(In)_X is equivalent to about 8.0, 9.0, 8.2 and 8.2 CO_2_ molecules per unit cell, respectively. Simultaneously, at 298 K and 1 atm (760 Torr), it corresponds to 5.6, 6.0, 5.1 and 5.0 CO_2_ molecules per unit cell (figure 6*d*). Considering the reductive surface area of the functionalized materials, the comparison result shows the –NH_2_ group has the obviously positive influence on the capacity of CO_2_ uptake. Besides –NH_2_, the insertion of the –NO_2_ group apparently enhances the CO_2_ adsorption capacity at low pressure, as shown in the inset of figure 6.

The *Q*_st_ of CO_2_ was studied to further understand the adsorption properties, which is determined by fits of the 273 K and 298 K sorption data. As shown in figure 5*b*, at the lowest coverage MIL-68(In)_NH_2_ indicates the highest value up to 24.3 kJ mol^−1^, and then followed by MIL-68(In)_NO_2_ (23.6 kJ mol^–1^) and MIL-68(In)_Br (20.0 kJ mol^–1^). All the values decrease gradually to 16.7 kJ mol^−1^ as the CO_2_ loading increases. However, the *Q*_st_ of MIL-68(In) is around 14.5 kJ mol^−1^ during the overall process, which effectively certifies that the polar organic groups are the favoured adsorption sites for CO_2_ adsorption and thus the insertion of these groups is an effectual approach to improve CO_2_ adsorption enthalpy. The higher value of MIL-68(In)_NO_2_ compared to MIL-68(In)_Br is in agreement with the greater attraction expected between the stronger polar functional group –NO_2_ and CO_2_, which has a significant quadrupole moment (table 2). With reference to the highest value of CO_2_ adsorption enthalpy of MIL-68(In)_NH_2_, it is believed to be generated from a combination of CO_2_–amine interactions, which is evidenced by other amino group modified MOF materials or computational studies [35–37]. During the CO_2_ coverage, the overlap of the favoured sites leads to gradual decrease in CO_2_ adsorption enthalpy.

To facilitate the contrast, the CO_2_ adsorption results of MIL-68(In) and MIL-68(In)_X are listed in table 5. CO_2_ uptake of the isomorphic MIL-68(In)_X is also affected by the surface area or pore volume at high pressure. However, the introduction of appropriate polar organic groups, outstandingly the –NH_2_ group, can effectively enhance the CO_2_ *Q*_st_, and apparently improve the CO_2_ adsorption capacity at low pressure.

**Table 5.** Results of CO_2_ adsorption measurement for MIL-68(In) and MIL-68(In)_X.

	273 K		298 K		*Q*_st_ (mmol g^–1^)
compound	mmol g^–1^	mole/unit cell	mmol g^–1^	mole/unit cell	
MIL-68(In)	2.25	8.0	1.58	5.6	14.7
MIL-68(In)_NH_2_	2.35	9.0	1.60	6.0	24.3
MIL-68(In)_Br	1.83	8.2	1.14	5.1	20.0
MIL-68(In)_NO_2_	1.92	8.2	1.22	5.0	23.6

## 4. Conclusion

We have demonstrated a series of crystalline structural transformations, among MIL-68(In)_X, MIL-53(In)_X or QMOF-2. It is notable to find the profound influence of the formula and the functional groups of the ligands on the product formation. It is proved that Ar with zero quadrupole moment is more suitable to probe the surface area of MIL-68(In)_X, in order to avoid the influence of the interactions of polar groups–N_2_ molecule. Ar adsorption result confirms that the influence of the size of the substituting groups on the surface area cannot be ignored. H_2_ and CO_2_ uptake of the MOF materials indicates that although the relative gas adsorption at high pressure is dominated by the surface, the appropriate grafted functions can effectively enhance the adsorption enthalpy, and apparently improve the gas adsorption capacity at low pressure. In contrast, the modification of –NO_2_ is best for H_2_ adsorption, while the grafted –NH_2_ is most beneficial to CO_2_ adsorption.

Based on the good porosity, remarkable stability and the existence of the polar sites in the framework, tentative further in-depth work on the functionalized MIL-68(In) materials will be undertaken in our laboratories to explore the other functional features.

## Supplementary Material

Supplementary Information

## Data accessibility

The datasets supporting this article have been uploaded as part of the electronic supplementary material.

## References

[1] Gygi D et al. 2016 Hydrogen storage in the expanded pore metal–organic frameworks M_2_(dobpdc) (M=Mg, Mn, Fe, Co, Ni, Zn). Chem. Mater. 28, 1128– 1138. (10.1021/acs.chemmater.5b04538)

[2] Witman M, Ling SL, Gladysiak A, Stylianou KC, Smit B, Slater B, Haranczyk M 2017 Rational design of a low-cost, high-performance metal–organic framework for hydrogen storage and carbon capture. J. Phys. Chem. C 121, 1171–1181. (10.1021/acs.jpcc.6b10363) PMC5253711 28127415

[3] Allen AJ, Espinal L, Wong-Ng W, Queen WL, Brown CM, Kline SR, Kauffman KL, Culp JT, Matranga C 2015 Flexible metal-organic framework compounds: *in situ* studies for selective CO_2_ capture. J. Alloys Compd. 647, 24–34. (10.1016/j.jallcom.2015.05.148)

[4] Wong-Ng W, Levin I, Kaduk JA, Espinal L, Wu H 2016 CO_2_ capture and positional disorder in Cu_3_(1,3,5-benzenetricarboxylate)_2_: an *in situ* laboratory X-ray powder diffraction study. J. Alloys Compd. 656, 200–205. (10.1016/j.jallcom.2015.09.078)

[5] Trickett CA, Helal A, Al-Maythalony BA, Yamani ZH, Cordova KE, Yaghi OM 2017 The chemistry of metal-organic frameworks for CO_2_ capture, regeneration and conversion. Nat. Rev. Mater. 2, 17045 (10.1038/natrevmats.2017.45)

[6] Ugale B, Dhankhar SS, Nagaraja CM 2016 Construction of 3-fold-interpenetrated three-dimensional metal–organic frameworks of nickel(II) for highly efficient capture and conversion of carbon dioxide. Inorg. Chem. 55, 9757–9766. (10.1021/acs.inorgchem.6b01569) 27649622

[7] Duan X, Wu C, Xiang SC, Zhou W, Yildirim T, Cui YJ, Yang Y, Chen BL, Qian GD 2015 Novel microporous metal–organic framework exhibiting high acetylene and methane storage capacities. Inorg. Chem. 54, 4377–4381. (10.1021/acs.inorgchem.5b00194) 25875579

[8] Lin JM, He CT, Liu Y, Liao PQ, Zhou DD, Zhang JP, Chen XM 2016 A metal–organic framework with a pore size/shape suitable for strong binding and close packing of methane. Angew. Chem. Int. Ed. 55, 4674–4678. (10.1002/anie.201511006) 26948156

[9] Alduhaish O, Wang H, Li B, Arman HD, Nesterov V, Alfooty K, Chen B 2016 A threefold interpenetrated pillared–layer metal–organic framework for selective separation of C_2_H_2_/CH_4_ and CO_2_/CH_4_. ChemPlusChem 81, 764–769. (10.1002/cplu.201600088) 31968817

[10] Sadakiyo M, Kuramoto T, Kato K, Yamauchi M 2017 Introduction of an amino group on zeolitic imidazolate framework through a ligand-exchange reaction. Chem. Lett. 47, 1004–1006. (10.1246/cl.170323)

[11] Munn AS, Pillai RS, Biswas S, Stock N, Maurin G, Walton RI 2016 The flexibility of modified-linker MIL-53 materials. Dalton Trans. 45, 4162–4168. (10.1039/C5DT03438H) 26465320

[12] Salah M, Marakchi K, Dalbouha S, Senent ML, Kabbaj OK, Komiha N 2015 Influence of the functionalization of imidazole on its CO_2_ uptake efficiency: a theoretical contribution. Comput. Theor. Chem. 1073, 1–8. (10.1016/j.comptc.2015.09.010)

[13] Xiong Y et al. 2016 Ligand and metal effects on the stability and adsorption properties of an isoreticular series of MOFs based on T-shaped ligands and paddle-wheel secondary building units. Chem. Eur. J. 22, 16 147–16 156. (10.1002/chem.201603299) 27699953

[14] Torrisi A, Mellot-Draznieks C, Bell RG 2010 Impact of ligands on CO_2_ adsorption in metal-organic frameworks: first principles study of the interaction of CO_2_ with functionalized benzenes. II. Effect of polar and acidic substituents. J. Chem. Phys. 132, 044705 (10.1063/1.3276105) 20113057

[15] Zhang YB et al. 2015 Introduction of functionality, selection of topology, and enhancement of gas adsorption in multivariate metal–organic framework-177. J. Am. Chem. Soc. 137, 2641–2650. (10.1021/ja512311a) 25646798

[16] Xia LZ, Liu Q, Wang FL, Lu JM 2016 Improving the hydrogen storage properties of metal-organic framework by functionalization. J. Mol. Model. 22, 254 (10.1007/s00894-016-3129-3) 27699551

[17] Frysali MG, Klontzas E, Tylianakis E, Froudakis GE 2016 Tuning the interaction strength and the adsorption of CO_2_ in metal organic frameworks by functionalization of the organic linkers. Micropor. Mesopor. Mater. 227, 144–151. (10.1016/j.micromeso.2016.02.045)

[18] Mu W, Liu DH, Yang QY, Chong LZ 2010 Computational study of the effect of organic linkers on natural gas upgrading in metal-organic frameworks. Micropor. Mesopor. Mater. 130, 76–82. (10.1016/j.micromeso.2009.10.015)

[19] Volkringer C et al. 2008 The Kagomé topology of the gallium and indium metal-organic framework types with a MIL-68 structure: synthesis, XRD, solid-state NMR characterizations, and hydrogen adsorption. Inorg. Chem. 47, 11 892–11 901. (10.1021/ic801624v) 19053340

[20] Perea-Cachero A, Romero E, Téllez C, Coronas J 2008 Insight into the reversible structural crystalline state transformation from MIL-53(Al) to MIL-68(Al). CrystEngComm. 20, 402–406. (10.1039/c7ce02034a)

[21] Carson F, Su J, Platero-Prats AE, Wan W, Yun YF, Samain L, Zou XD 2013 Framework isomerism in vanadium metal–organic frameworks: MIL-88B(V) and MIL-101(V). Cryst. Growth Des. 13, 5036–5044. (10.1021/cg4012058)

[22] Liédana N, Lozano P, Galve A, Téllez C, Coronas J 2014 The template role of caffeine in its one-step encapsulation in MOF NH_2_-MIL-88B(Fe). J. Mater. Chem. B 2, 1144–1151. (10.1039/c3tb21707h) 32261350

[23] Anokhina EV, Vougo-Zanda M, Wang XQ, Jacobson AJ 2005 In(OH)BDC·0.75BDCH_2_ (BDC=benzenedicarboxylate), a hybrid inorganic-organic vernier structure. J. Am. Chem. Soc. 127, 15 000–15 001. (10.1021/ja055757a) 16248619

[24] Sun JY, Weng LH, Zhou YM, Chen JX, Chen ZX, Liu ZC, Zhao DY 2002 QMOF-1 and QMOF-2: three-dimensional metal-organic open frameworks with a quartzlike topology. Angew. Chem. Int. Ed. 41, 4471–4473. (10.1002/1521-3773(20021202)41:23%3C4471::AID-ANIE4471%3E3.0.CO;2-9) 12458509

[25] Choi S, Lee HJ, Kim T, Oh M 2014 Structural and morphological transformations of In–MIL-68-based hexagonal lumps to QMOF-2-based pointed hexagonal rods by means of destruction and reconstruction processes. Eur. J. Inorg. Chem. 2014, 6220–6224. (10.1002/ejic.201402905)

[26] Boultif A, Louër D 2004 Powder pattern indexing with the dichotomy method. J. Appl. Crystallogr. 37, 724–731. (10.1107/S0021889804014876)

[27] Li JR, Kuppler RJ, Zhou HC 2009 Selective gas adsorption and separation in metal-organic frameworks. Chem. Soc. Rev. 38, 1477–1504. (10.1039/b802426j) 19384449

[28] Banerjee R, Furukawa H, Britt D, Knobler C, O'Keeffe M, Yaghi OM 2009 Control of pore size and functionality in isoreticular zeolitic imidazolate frameworks and their carbon dioxide selective capture properties. J. Am. Chem. Soc. 131, 3875–3877. (10.1021/ja809459e) 19292488

[29] Garibay SJ, Cohen SM 2010 Isoreticular synthesis and modification of frameworks with the UiO-66 topology. Chem. Commun. 46, 7700–7702. (10.1039/C0CC02990D) PMC3581306 20871917

[30] Mowat JPS et al. 2011 Structural chemistry, monoclinic-to-orthorhombic phase transition, and CO_2_ adsorption behavior of the small pore scandium terephthalate, Sc_2_(O_2_CC_6_H_4_CO_2_)_3_, and its nitro- and amino-functionalized derivatives. Inorg. Chem. 50, 10 844–10 858. (10.1021/ic201387d) 21958382

[31] Rudzinski W, Everett DH 1992 Adsorption of gases on heterogeneous surfaces. London, UK: Academic Press.

[32] Jaroniek M, Madey R 1988 Physical adsorption on heterogeneous solids. Amsterdam, The Netherlands: Elsevier.

[33] Kaye SS, Dailly A, Yaghi OM, Long JR 2007 Impact of preparation and handling on the hydrogen storage properties of Zn_4_O(1,4-benzenedicarboxylate)_3_ (MOF-5). J. Am. Chem. Soc. 129, 14 176–14 177. (10.1021/ja076877g) 17967030

[34] Hübner O, Glöss A, Fichtner M, Klopper W 2004 On the interaction of dihydrogen with aromatic systems. J. Phys. Chem. A 108, 3019–3023. (10.1021/jp031102p)

[35] Demessence A, D'Alessandro DM, Foo ML, Long JR 2009 Strong CO_2_ binding in a water-stable, triazolate-bridged metal-organic framework functionalized with ethylenediamine. J. Am. Chem. Soc. 131, 8784–8786. (10.1021/ja903411w) 19505094

[36] McDonald TM, D'Alessandro DM, Krishnac R, Long JR 2011 Enhanced carbon dioxide capture upon incorporation of N,N’-dimethylethylenediamine in the metal-organic framework CuBTTri. Chem. Sci. 2, 2022–2028. (10.1039/c1sc00354b)

[37] Panda T, Pachfule P, Chen YF, Banerjee R 2011 Amino functionalized zeolitic tetrazolate framework (ZTF) with high capacity for storage of carbon dioxide. Chem. Commun. 47, 2011–2013. (10.1039/C0CC04169F) 21180716

