# Supplementary material for: Impact of the functionalization onto structure transformation and gas adsorption of MIL-68(In)
Source: R Soc Open Sci. 2018 Dec 12;5(12):181378. doi: 10.1098/rsos.181378 (PMC6304125; doi:10.1098/rsos.181378)
Supplement: Supplementary Information [file rsos181378supp1.docx]

Supporting information for

# Impact of the functionalization onto structure transformation and gas adsorption of MIL-68(In)

Lei Wu*^ab^, Weifeng Wang ^ab^, Rong Liu ^ab^, Gang Wu^c^ and Huaxin Chen^b^

*^a^* Polymer Materials & Engineering Department, School of Materials Science & Engineering, Chang’an University, Xi’an 710064, China

*^b^* Engineering Research Center of Transportation Materials Ministry of Education, Chang’an University, Xi'an 710064, China

*^c^* State Key Laboratory of Inorganic Synthesis and Preparative Chemistry, College of Chemistry, Jilin University, Changchun 130012, P.R. China.

To whom correspondence should be addressed. E-mail: [wulei@chd.edu.cn](mailto:wulei@chd.edu.cn); Tel: +86 29 82337258.


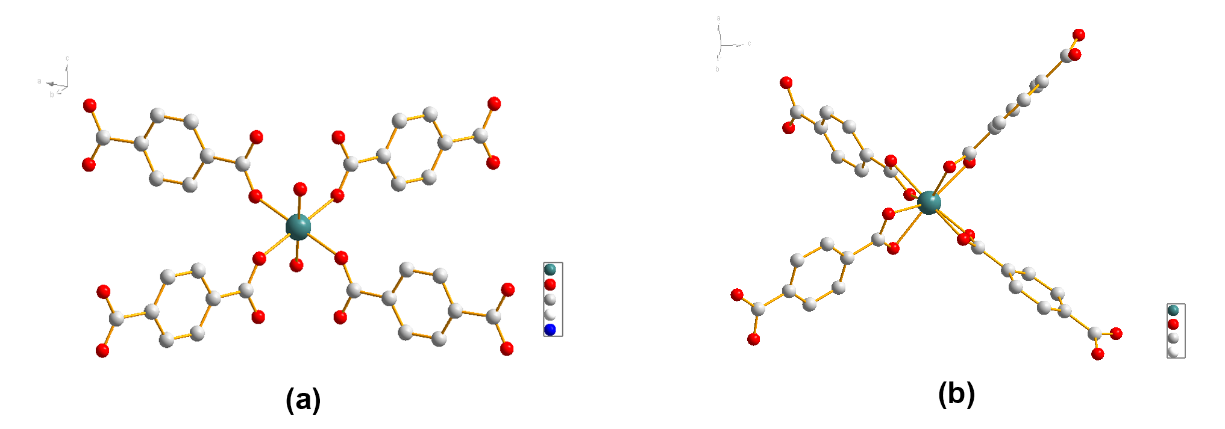


**Figure S1** Coordination environment of In^3+^ of (a) MIL-68(In) and MIL-53(In) ; (b) QMOF-2 (Color code: In, olive; C, French grey; O, red) .





**Figure S2** PXRD patterns for (a) MIL-68(In) simulated from single–crystal structure analysis; (b) MIL-68(In)_NH_2_; (c) MIL-68(In)_Br; (d) MIL-68(In)_NO_2_.

### *Infrared Spectroscopy*

The IR spectra of as-synthesized MIL-68(In)­_X samples are investigated as shown in Figure S3. For comparison, the one of as-synthesized MIL-68(In)­ sample are investigated as well. All IR spectrum of as-synthesized samples exhibits the typical vibrational bands of the carboxylic acid function in the region of 1400-1700 cm^-1^. The adsorption band of the carboxyl groups of the ligand coordinated to the metal centers is visible at 1558 cm^-1^ whereas the one of pure ligand is observed at 1693 cm^-1^. Such difference proves the absence of free ligand in the as-synthesized samples. Moreover, the presence of occluded DMF molecules is also evidenced by its C=O band which appears at around 1660 cm^-1^. The broad peak between 3600 and 2500 cm^-1^ is mainly due to water molecules and the *μ*–OH of the network.

The peak at 1256 cm^-1^ corresponds to the stretching vibration of N-C and the two weak peaks at 3380 and 3472 cm^-1^ correspond to symmetric and asymmetric stretching vibrations of NH_2_ species of the ligand, respectively. The peak at 1039 cm^-1^ corresponds to the stretching vibration of Br-C and the two weak peaks at 1497 and 1304 cm^-1^ correspond to stretching vibrations of O-N and N-C for NO_2_ species, respectively. The appearance of these signals in the spectrum of MIL-68(In)_X showing the incorporation of the ligand into the framework with the uncoordinated functionalization species.





**Figure S3** The IR spectra for the activated samples of (a) MIL-68(In); (b) MIL-68(In)_NH_2_; (c) MIL-68(In)_Br; (d) MIL-68(In)_NO_2_ (●, ν_s(N–H)_ ; ●, ν_as(N–H)_; ●, ν_(C–N)_; *, ν_s(C–Br)_; ♦, ν_(N–O)_; ♦, ν_(C–N)_).

### Stability Analysis of MIL-68(In)_X

TGA and PXRD were performed to investigate the stability of MIL-68(In)_X.

The TG curves of the as-synthesized MIL-68(In)_X samples show three similar weight losses as the as-synthesized MIL-68(In) sample (Figure S4). The first two weight losses from room temperature to 200 °C indicated the existence of the trapped trapped water and occluded DMF molecules. The framework remains stable till around 400 °C, then collapses and transforms into In_2_O_3_. The TGA was also performed on the activated samples. The first two weight losses corresponding to the trapped solvent molecules in the crude samples are vanished in all the TG curves. This obvious difference indicates the efficient removal of all the guest molecules in the pores of the as-synthesized samples.


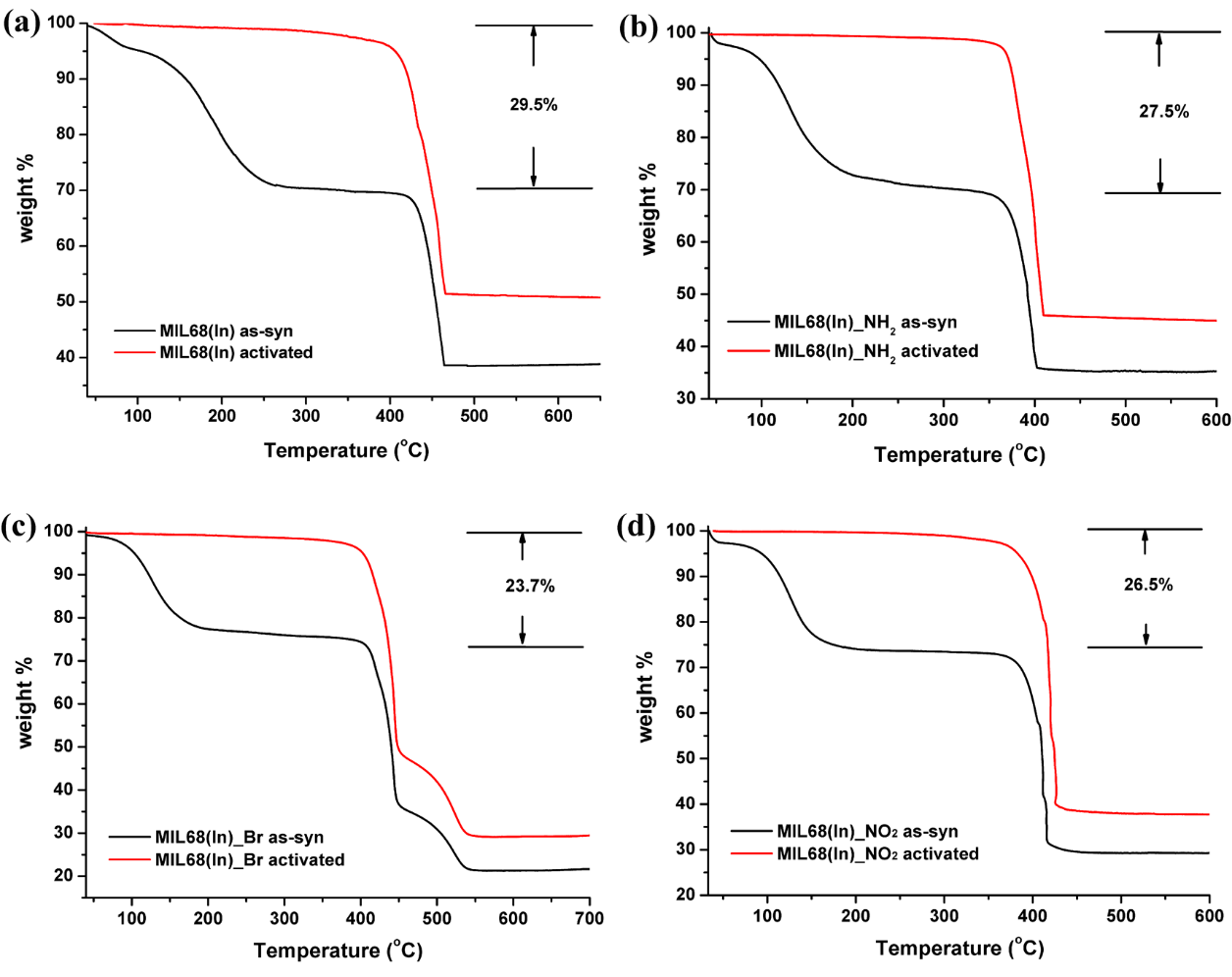


**Figure S4** The TGA curves of (a) MIL-68(In); (b) MIL-68(In)_NH_2_; (c) MIL-68(In)_Br; (d) MIL-68(In)_NO_2_ (as-synthsized samples, black; activatedsamples, red).

Simultaneously, seen from PXRD patterns of the as-synthsized samples, activated samples and the samples after N_2_ adsorption measurement, the good agreement indicates the perfect sustainment of the whole framework afterwards activation process and gas adsorption measurement (Figure S5).


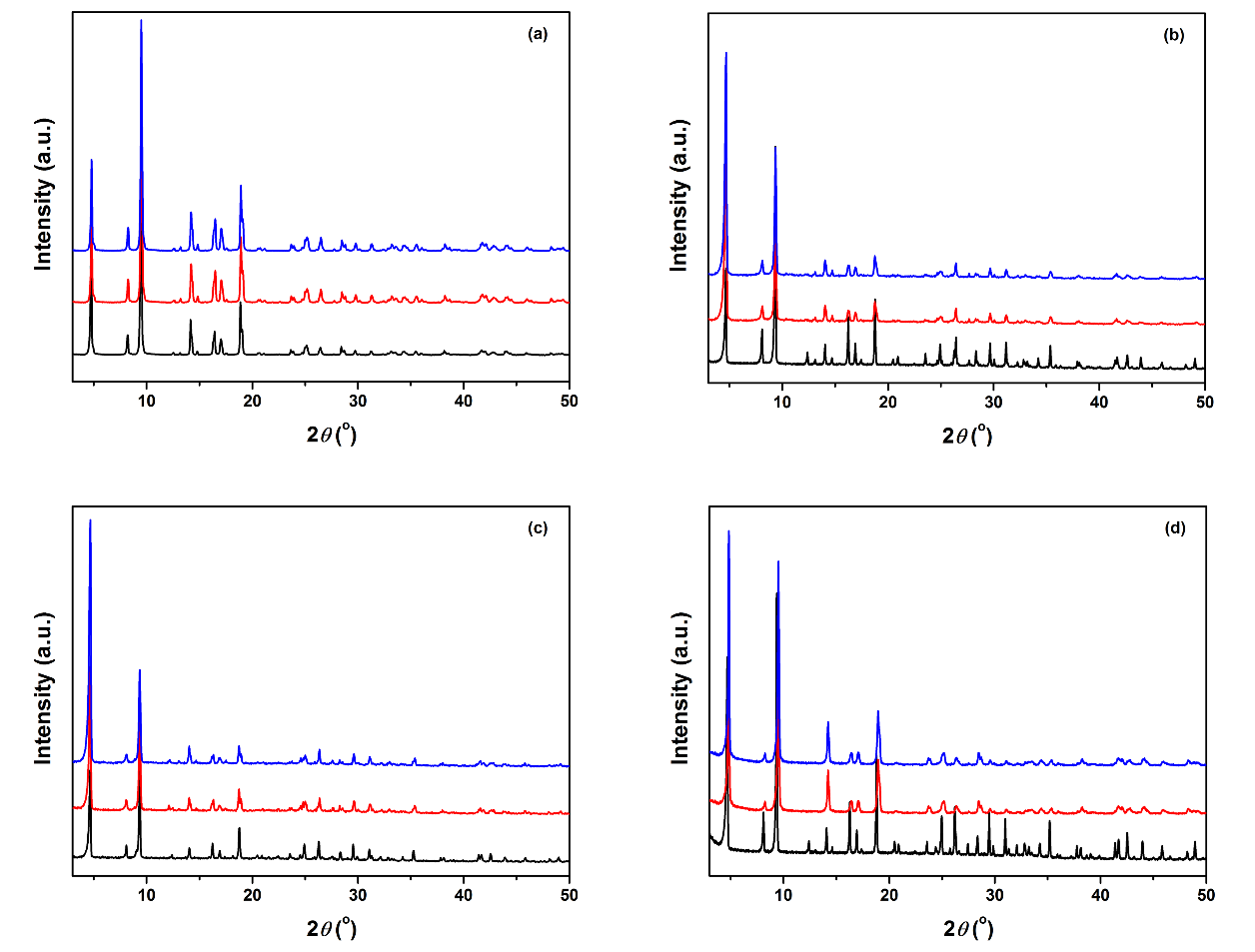


**Figure S5** The PXRD patterns for (a) MIL-68(In); (b) MIL-68(In)_NH_2_; (c) MIL-68(In)_Br; (d) MIL-68(In)_NO_2_ (as-synthsized samples, black; activated samples, red; samples after N_2_ adsorption, blue).

### ^1^HNMR Spectroscopy Analysis of MIL-68(In)_X

The ^1^H NMR spectra of pure ligands accompanied with corresponding as-synthesized and activated samples of MIL-68(In)_X are depicted in Figure S6. All of the spectra display three distinct sets of signals assignable to the phenyl protons of each ligand (^1^HNMR (400 MHz, DCl/D_2_O/DMSO-d_6_) H_2_BDC-NH_2_: *δ*7.87 (d, 1H, ^3^J= 8.31 Hz); 7.66 (s, 1H); 7.36 (d, 1H, ^3^J= 8.31 Hz); H_2_BDC-Br: *δ*7.79 (d, 1H, ^3^J= 8.06 Hz), 7.95 (d, 1H, ^3^J= 8.06 Hz), 8.11 (s, 1H); H_2_BDC-NO_2_: *δ*8.36 (s, 1H), 8.26 (d, 1H, ^3^J= 7.81 Hz), 7.92 (d, 1H, ^3^J= 7.81 Hz)). Three additional signals corresponding to DMF molecules are clearly evidenced on the as-synthesized MIL-68(In)_X spectra. The disappearance of DMF signals in the ^1^H NMR spectrum of the activated MIL-68(In)_X samples confirms the effectiveness of the activation method to remove trapped DMF molecules.


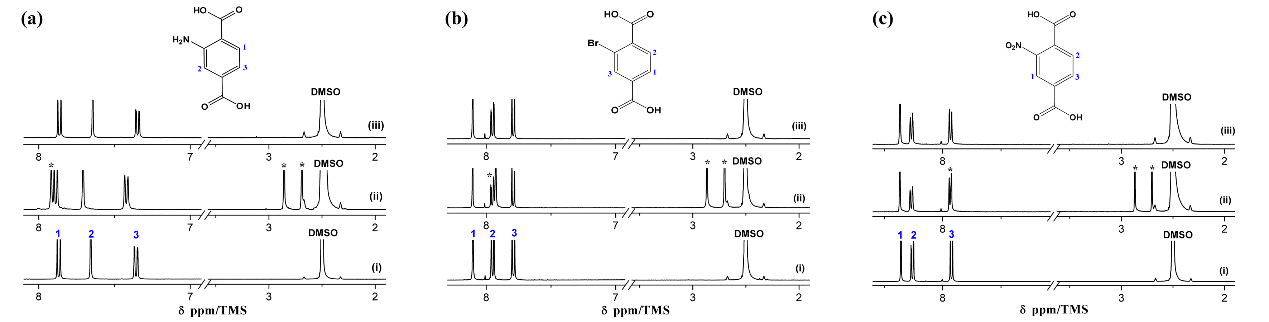


**Figure S6** ^1^H NMR spectra of (a) (i) H_2_BDC-NH_2_ ligand; (ii) as-synthesized; (iii) activated MIL-68(In)_NH_2_; (b) (i) H_2_BDC-Br ligand; (ii) as-synthesized; (iii) activatedMIL-68(In)_Br; (c) (i) H_2_BDC-NO_2_ ligand; (ii) as-synthesized; (iii) activated MIL-68(In)_NO_2_ (* marks denote the signals belonging to DMF).


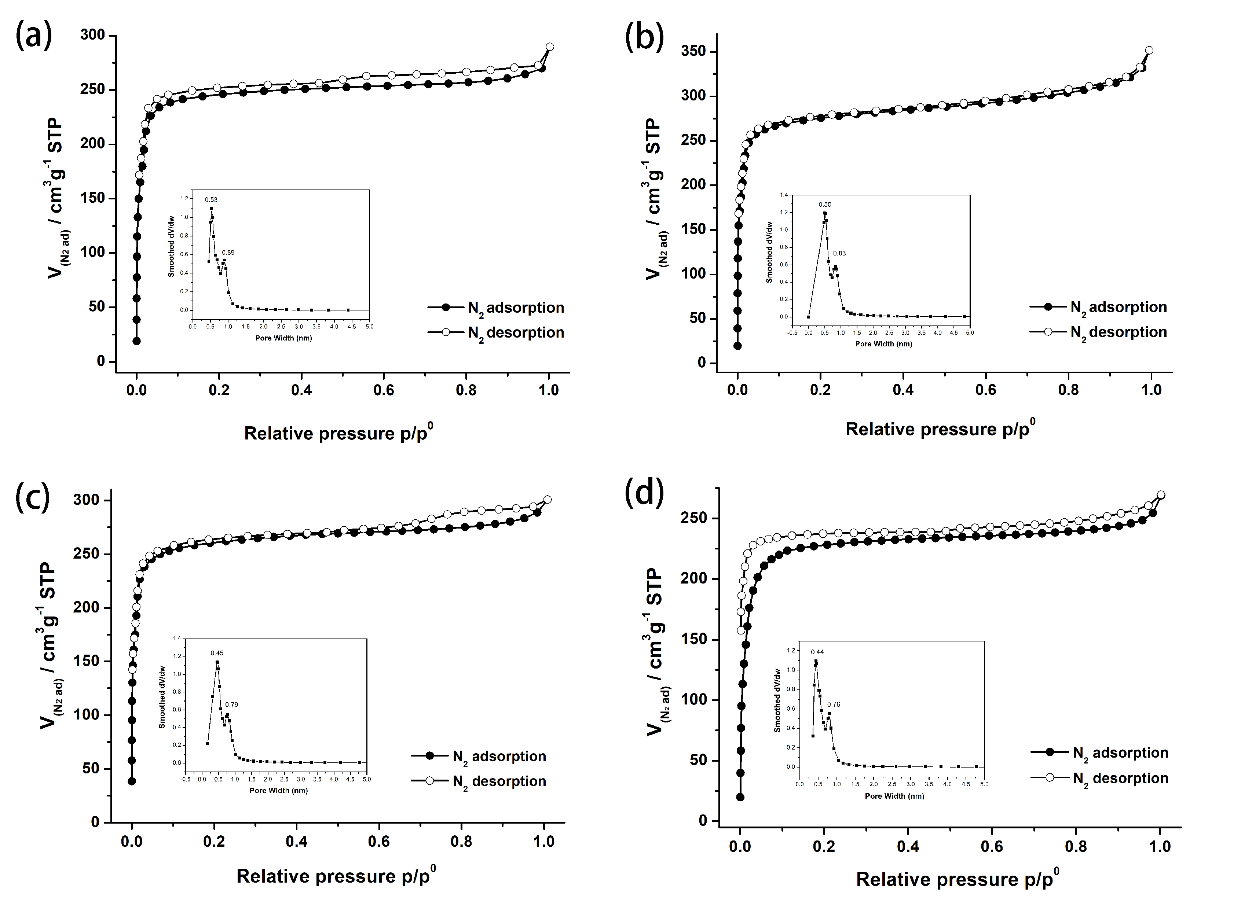


**Figure S7** At 77 K, the N_2_ sorption isotherms of (a) MIL-68(In); (b) MIL-68(In)_NH_2_; (c) MIL-68(In)_Br; (d) MIL-68(In)_NO_2_ (adsorption, solid; desorption, empty). The insets are the pore size distributions.


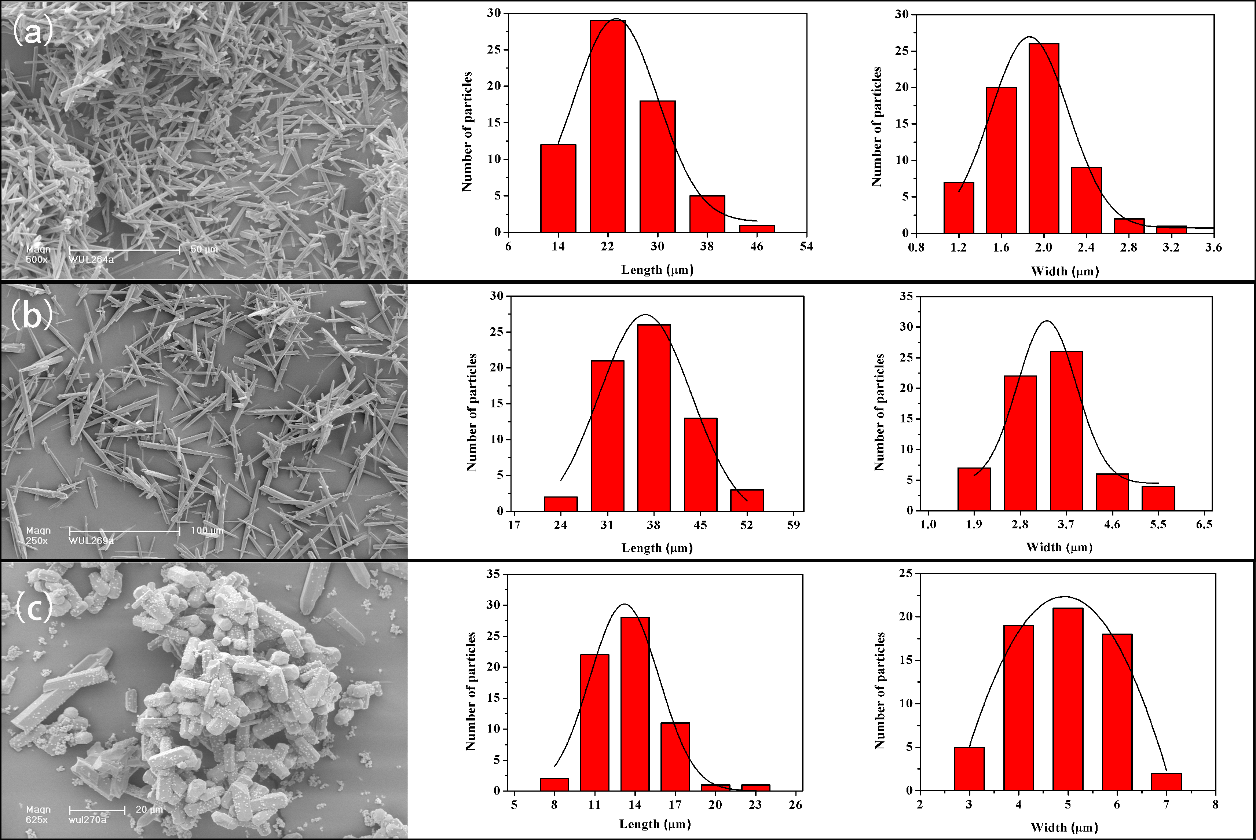


**Figure S8** SEM photographs and the histograms of the size distribution of the samples: (a) MIL-68(In)_NH_2_; (b) MIL-68(In)_Br; (c) MIL-68(In)_NO_2_.
